# Temporal dysregulation of the somatomotor network in agitated depression

**DOI:** 10.1093/braincomms/fcae425

**Published:** 2024-11-26

**Authors:** Qunjun Liang, Ziyun Xu, Shengli Chen, Shiwei Lin, Xiaoshan Lin, Ying Li, Yingli Zhang, Bo Peng, Gangqiang Hou, Yingwei Qiu

**Affiliations:** Department of Radiology, Shenzhen Nanshan People’s Hospital, Shenzhen University, Shenzhen 518000, People’s Republic of China; Guangdong Key Laboratory for Biomedical Measurements and Ultrasound Imaging, National-Regional Key Technology Engineering Laboratory for Medical Ultrasound, School of Biomedical Engineering, Shenzhen University Medical School, Shenzhen 518060, People’s Republic of China; Department of Radiology, Shenzhen Kangning Hospital, Shenzhen Mental Health Center, Shenzhen 518020, People’s Republic of China; Department of Radiology, Shenzhen Nanshan People’s Hospital, Shenzhen University, Shenzhen 518000, People’s Republic of China; Department of Radiology, Shenzhen Nanshan People’s Hospital, Shenzhen University, Shenzhen 518000, People’s Republic of China; Department of Radiology, Shenzhen Nanshan People’s Hospital, Shenzhen University, Shenzhen 518000, People’s Republic of China; Department of Radiology, Shenzhen Nanshan People’s Hospital, Shenzhen University, Shenzhen 518000, People’s Republic of China; Department of Depression, Shenzhen Kangning Hospital, Shenzhen Mental Health Center, Shenzhen 518020, People’s Republic of China; Department of Depression, Shenzhen Kangning Hospital, Shenzhen Mental Health Center, Shenzhen 518020, People’s Republic of China; Department of Radiology, Shenzhen Kangning Hospital, Shenzhen Mental Health Center, Shenzhen 518020, People’s Republic of China; Department of Radiology, Shenzhen Nanshan People’s Hospital, Shenzhen University, Shenzhen 518000, People’s Republic of China

**Keywords:** agitated depression, time delay estimation, edge-centre time series, functional magnetic resonance

## Abstract

Agitated depression (A-MDD) is a severe subtype of major depressive disorder, with an increased risk of suicidality and the potential to evolve into bipolar disorder. Despite its clinical significance, the neural basis remains unclear. We hypothesize that psychomotor agitation, marked by pressured speech and racing thoughts, is linked to disruptions in brain dynamics. To test this hypothesis, we examined brain dynamics using time delay estimation and edge-centre time series, as well as dynamic connections between the somatomotor network (SMN) and the default mode network in 44 patients with A-MDD, 75 with non-agitated MDD (NA-MDD), and 94 healthy controls. Our results revealed that the neural co-activity duration was shorter in the A-MDD group compared with both the NA-MDD and controls (A-MDD versus NA-MDD: *t* = 2.295; A-MDD versus controls: *t* = 2.192, all *P* < 0.05). In addition, the dynamic of neural fluctuation in SMN altered in the A-MDD group than in the NA-MDD group (*t* = −2.616, *P* = 0.011) and was correlated with agitation severity (*β* = −0.228, *P* = 0.011). The inter-network connection was reduced in the A-MDD group compared with the control group (*t* = 2.102, *P* = 0.037), especially at low-amplitude time points (*t* = 2.139, *P* = 0.034). These findings indicate rapid neural fluctuations and disrupted dynamic coupling between the SMN and default mode network in A-MDD, potentially underlying the psychomotor agitation characteristic of this subtype. These insights contribute to a more nuanced understanding of the heterogeneity of depression and have implications for differential diagnosis and treatment strategies.

## Teaser

Agitated depression, a subtype of clinical depression, exhibits heightened agitation. Liang *et al*. used functional MRI to show that this subtype has an accelerated network transition rate, earlier somatomotor network delay, and reduced dynamic connectivity between the somatomotor and default mode networks, which also correlates with patient’s agitation severity.

## Graphical Abstract

**Graphical abstract:**
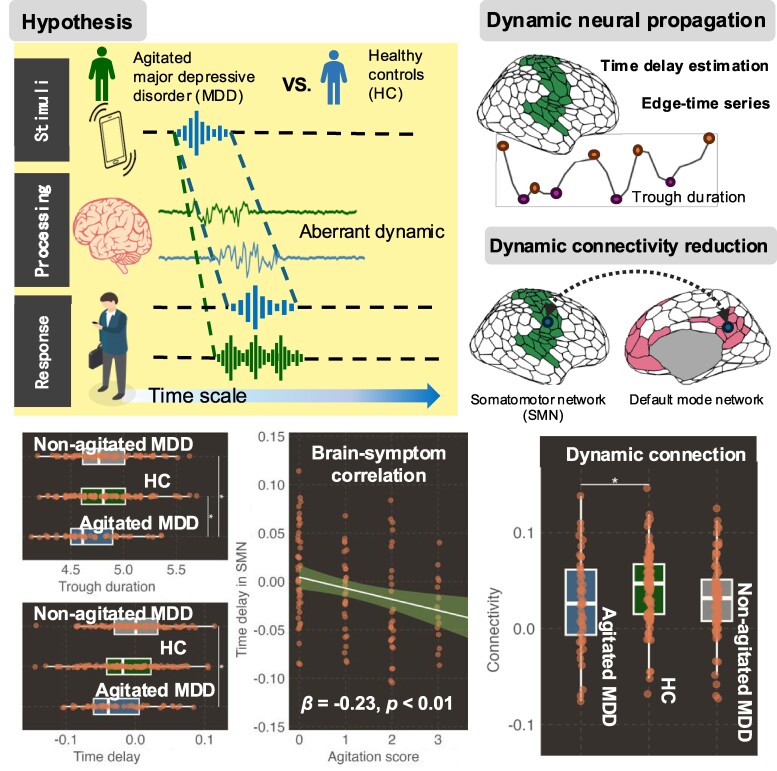

## Introduction

Depressive disorders are a pervasive mental illness affecting an estimated 300 million individuals globally.^1^ However, the clinical presentation of major depressive disorder (MDD) is heterogeneous.^2^ Psychomotor agitation, a type of psychomotor disturbance, is marked by unconscious movements stemming from mental tension, such as restlessness, incessant talking, and fidgeting.^3^ MDD patients exhibiting agitation are classified as an agitated subtype (A-MDD).^4^ In addition to psychomotor agitation, patients with A-MDD also present with elevated levels of both depression and anxiety, resulting in a severe subtype of clinical depression.^5^ A previous study found that patients with A-MDD demonstrated a heightened propensity for behavioural problems, like substance abuse,^3^ and have elevated risks of mania, which can lead to the transition to bipolar disorder (BD).^6,7^ Despite these associations, the neural basis underlying A-MDD remains elusive.

**Figure 1:**
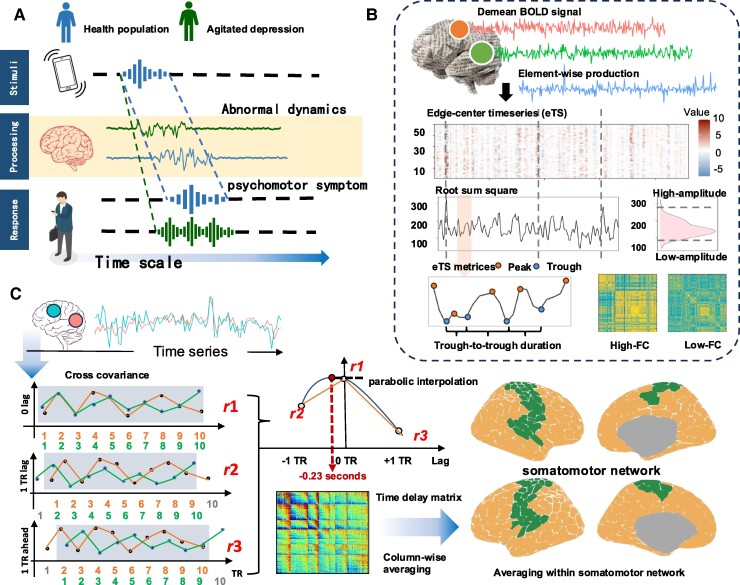
**Schematic illustration of the hypothesis and dynamic measurements. A.** Scheme of the link between agitation and aberrant neuronal fluctuation in our hypothesis. Patients with A-MDD interact with the environment in an excited and irritated type, which may reflect abnormal neuronal fluctuation. **B**. Assessing the whole-brain co-fluctuation with edge-centre time series (eTS). Two metrics of temporal properties, trough-to-trough duration and peak height, were generated from the eTS. Two time-varying FCs, high- and low-amplitude FCs, were also obtained from the eTS. **C**. Time delay (TD) estimation, which reflects the relative sequence of specific areas in whole-brain propagation. TD in the SMN (in green) was extracted from the TD projection map. Abbreviations: TR, repetition time; BOLD, blood oxygen level-dependent.

Agitation, characterized by excessive and rapid movements, may be linked to aberrant dynamics in neuronal fluctuations. Research in healthy populations has shown that neural oscillations synchronize with environmental stimuli, thereby facilitating information processing^8^ and actively shaping perception.^9^ These findings suggest a temporal alignment between neuronal activity and external stimuli (physical stimuli). In the field of mental health, spatiotemporal psychopathology (STPP) posits that the pathogenesis of agitation stems from a desynchronization between the ‘internal’ neural activity and the ‘external’ world (Fig. 1A).^10,11^ This intrinsic alteration in the patient’s internal time scale can be discerned through the temporal features of neural spontaneous activity.^12,13^ For instance, neuronal variability, as measured by resting-state functional magnetic resonance imaging (fMRI), has demonstrated significant differences among patients with BD during various episodes of the disorder.^14^ Moreover, research indicates that rsfMRI can predict the task performance^15^ and behavioural traits,^16^ implying that the spontaneous neural activity may serve as an indicator of the baseline deviance associated with psychiatric risk factors.

The somatomotor network (SMN) is pivotal in movement disorders. The SMN encompasses the primary motor cortex (MI), the caudal premotor area, and the supplemental motor area, all of which contribute to sensory integration and modulate interactions with the external environment.^17^ SMN dysfunction, particularly aberrations in its dynamic properties, has been implicated in various motor-related brain diseases, like attention-deficit/hyperactivity disorder^18^ and Parkinson’s disease.^19^ A previous study reported that the disruption of the connection between the SMN and a non-motor cortical network, like the default mode network (DMN), was associated with psychomotor disturbances in BD.^20^ These findings suggest that studies of the neural mechanisms related to agitation in depressive disorders should consider not only SMN activity itself but also the connectivity between the SMN and the DMN.

The present study was aimed at characterizing the neural underpinnings related to psychomotor agitation in MDD. By assessing edge-centre time series (eTS)^21^ and time delay (TD) estimation^22^ as metrics for brain dynamics, we tested the following hypotheses: (i) Brain dynamics differ in patients with A-MDD compared with both healthy controls (HCs) and patients with non-agitated MDD (NA-MDD); (ii) these alterations are especially pronounced within the SMN; and (iii) the connectivity between the SMN and the DMN is diminished in patients with A-MDD relative to healthy populations.

## Materials and methods

### Participants

The current study enrolled 151 patients diagnosed with MDD according to the Diagnostic Statistical Manual of Mental Disorders, Fifth Edition (DSM-5) criteria, and 107 HCs, based on the following inclusion criteria: (i) aged 18 years or older, (ii) the absence of significant prior neurological conditions, such as stroke or substantial head trauma, and (iii) no contraindications for MRI scanning (Supplementary Table 1). The patients with MDD were inpatients from the Department of Depression at Shenzhen Kangning Hospital. HCs were recruited from the hospital staff and the local community, all of whom were free of psychiatric diagnoses. Both the 17-item Hamilton Depression Rating Scale (HAMD) and the Hamilton Anxiety Rating Scale (HAMA) were administered during the first week of hospitalization. Patients with HAMD scores below 17 were excluded from the analysis. This study was conducted with the approval of the Institutional Review Board of Shenzhen Kangning Hospital.

### Agitated depression identification

Referring to a previous study,^23^ we categorized patients with MDD with noticeable agitation symptoms into the A-MDD subgroup. Patients with moderate agitation or above (HAMD item 9 score equal to or more than 2, Supplementary Fig. 1) were also classified into the A-MDD subgroup, while the other patients were classified into the NA-MDD subgroup. Because the HAMD was evaluated during the first week of the patient’s hospitalization, we also excluded the confounder of secondary agitation induced by the side effects of antidepressant use.^24^

### MRI data acquisition

MRI data were obtained using a 3.0 Tesla scanner (Discovery MR750 System; General Electric, Milwaukee, WI, USA) with an eight-channel head coil at Shenzhen Kangning Hospital. All participants were instructed to remain still, stay awake with their eyes closed, and not think about anything. The fMRI data were obtained using an echo-planar imaging sequence with the following parameters: repetition time (TR) = 2000ms, echo time (TE) = 30 ms, flip angle = 90°, thickness/gap = 3.5/0.7 mm, acquisition matrix = 64 × 64, field of view (FOV) = 224 mm^2^, 33 axial slices, and 240 time points (8 min). In addition, high-resolution structural MRI scans were obtained using a fast-field echo three-dimensional T1-weighted (3D-T1WI) sequence. The details parameters were as follows: TR/TE = 6.65/2.93 ms, flip angle = 12°, acquisition matrix = 256 × 256, FOV = 256 × 256 mm^2^, and 192 sagittal slices with no inter-slice gap.

### Functional data preprocessing

We performed quality control on the images using MRIQC ver. 22.0.6 before image preprocessing. We implemented stringent criteria for head motion according to Mitra *et al*.^25^ to ensure the accuracy of the dynamic estimations. The evaluation of head motion was based on frame-wise displacement (FD), where (i) a mean FD value greater than 0.25 mm and (ii) the presence of spike volumes above 25% of the total times series were considered indicative of excessive motion. Consequently, fMRI data from 17 patients with MDD and 13 HCs were excluded based on these head-motion criteria.

The functional images were preprocessed via fMRIPrep ver. 22.1.1.^26^ Briefly, the steps included (i) slice-timing correction, (ii) head-motion correction with six parameters, (iii) alignment between functional images and T1W1 images, and (iv) spatial normalization to the MNI standard space. After preprocessing, brain signal time series were extracted using the Power-264 atlas,^27^ regressing out the head-motion parameters, detrending with a bandpass of 0.01–0.1 Hz, and smoothing for a 6 mm full-width half-maximum kernel. Regression for the global signal was not performed to ensure the precision of the dynamic metric calculations.^28^

We excluded the subcortical and cerebellum regions in the Power-264 atlas by referring to a previous study,^29^ which resulted in 214 parcels. Nuisance regression and blood oxygen level-dependent (BOLD) signal extraction were performed using the Python package Nilearn (https://nilearn.github.io/dev/index.html).

### Edge-centre time series

eTS focuses on the whole-brain co-fluctuation pattern, which indicates the temporal properties of brain activity (Fig. 1B).^28^ BOLD signals with length *T* of parcels $x_{i}$ and $x_{j}$ were *z*-scored first. Then, the element-wise product of time series $x_{i}$ and $x_{j}$ was calculated. This procedure was repeated for all pairs of 214 parcels, and an eTS matrix with dimensions $\left[\frac{214\times (214-1)}{2}\times T\right]$ was obtained. Finally, the root sum square (RSS) was estimated by columns in the eTS matrix, resulting in a vector of length *T*, which reflected the whole-brain co-fluctuations in the time scale.

The dynamic throughout the eTS for each participant was calculated as the mean duration and RSS. Duration was measured by the frames (units in TR) between two troughs in the RSS, while the mean duration was the average of all durations, representing the temporal properties of the eTS.^19^ For a given time series length, an RSS with a shorter mean duration reflects faster neuronal fluctuation from the macro level.

As a comparison, we also evaluated the peak height from the RSS, which provided the average peak amplitude in the time series. eTS was acquired using the MATLAB script provided by Zamani *et al*.^21^

### Time delay estimation

The time shifts in SMN were calculated using TD, which exhibited stereotypical propagation patterns across the brain (Fig. 1C).^22^ The TD computation was based on the cross-covariance function (CCF) between two time series $x_{i}(T)$ and $x_{j}(T)$. In practice, CCF was applied to the shifted time series as


$$c_{x_{i}x_{j}}(\Delta )=x_{i}(T+\Delta )\cdot x_{j}(T),$$

where Δ is the shifted interval corresponding to TR units, like [TR-2, TR-1, TR, TR + 1, TR + 2]. Time delay (${\hat{\tau }}_{1,2}$) is then estimated by a three-point parabolic interpolation on the peak of $c_{x_{i}x_{j}}(\Delta )$ as


$${\hat{\tau }}_{1,2}=TR\frac{c_{\mathrm{peak}-1}-c_{\mathrm{peak}+1}}{2(c_{\mathrm{peak}-1}-2c_{\mathrm{peak}}+c_{\mathrm{peak}+1})},$$

where $c_{\mathrm{peak}}$ is the peak among $c_{x_{1}x_{2}}(\Delta )$ and the values immediately preceding ($c_{\mathrm{peak}-1}$) and succeeding ($c_{\mathrm{peak}+1}$).

The above approach was applied to each pair of the 214 parcels, resulting in a 214 × 214 TD matrix. Then, a time delay projection map was generated by column-wise averaging of the TD matrix, resulting in a vector with 214 elements. Finally, we extracted the TD of the SMN by averaging the SMN-related elements (34 regions of interest, ROIs) in the TD projection map. We performed TD estimation with in-house scripts, adjusting to the script provided by Raut *et al*.^30^

### Inter-network functional connectivity

Static and time-varying SMN–DMN connectivity was obtained using static FC and eTS-based FC approaches. The static FC matrix was derived by calculating Pearson’s correlation across parcels within the SMN and the DMN (57 ROIs). Subsequently, a static SMN–DMN connection was obtained by averaging the inter-network FC in the static FC matrix. For high- and low-amplitude connections, the time points of the top and bottom 10% peaks/troughs in the RSS were identified first. Then, the SMN–DMN connection was obtained as the approach of static FC restricted to the high- and low-amplitude time points. In a previous study, the eTS-based FC was shown to represent time-varying fluctuations in brain state.^31^ Thus, by employing eTS-based FC, our goal was to dissect the contributions of time-varying SMN–DMN co-fluctuations to static FC dysfunction in A-MDD.

### Statistical analysis

#### Clinical profile description

The demographic variables and HAMD and HAMA total scores were compared between the A-MDD and NA-MDD groups using the independent *t*-test. Differences in specific symptoms were further analyzed by comparing the scores of all 17 items of the HAMD-17 between the two MDD subgroups using one-way ANOVA, followed by a post-hoc comparison for each item. The *P*-values in the post-hoc comparison were corrected using the false discovery rate (FDR) method. We presented both the uncorrected *P*-values and the corrected *P*-values to provide comprehensive insight into the results.

#### Exploring alterations in brain dynamics

The alterations in brain dynamics were elucidated by assessing the mean trough duration and TD in the SMN in the A-MDD, NA-MDD, and HC groups. Given the differing covariates, three independent *t*-tests were conducted: A-MDD versus HC, A-MDD versus NA-MDD, and NA-MDD versus HC. For these comparisons, we controlled for age, gender, and education level to mitigate confounding effects. In addition, for comparisons between A-MDD and NA-MDD, the total HAMA score and the HAMD depression sub-dimension score^32^ were incorporated as covariates. The *P*-values obtained from these *t*-tests were adjusted using the FDR method to correct for multiple comparisons. Peak height in the eTS was examined in the three groups by employing the same analytical approach to determine whether the intensity of brain activity was also modified in patients with A-MDD.

Two linear regression models were built to examine the correlation between trough duration and agitation and TD in SMN and agitation. The models took the brain metric as the predictor and agitation scores as the dependent variable, controlling for gender, age, education level, and anxiety level (HAMA total score). All variables were centralized before projection to the model in case of multicollinearity.

#### SMN–DMN connectivity changes in A-MDD

In our hypothesis, agitation MDD would exhibit an atypical connection between the SMN and the DMN. This hypothesis was validated by employing *t*-tests to test for differences in static FC between the A-MDD, NA-MDD, and HC groups. We conducted comparative analyses of high- and low-amplitude FC between the two groups to determine whether the dynamic aspects of SMN–DMN connectivity played a role in the observed alterations. In all *t*-tests, we rigorously controlled for potential confounding variables, such as gender, age, and education level. HAMA scores and depression severity were also controlled for between the MDD subgroups. The FDR method was applied to correct for multiple comparisons to ensure the robustness of our findings.

#### Control analyses

Two control analyses were performed to demonstrate the specificity of the effects in the agitation subtype. First, we examined the impact of varying anxiety levels on brain dynamics by re-classifying the patients into severe and moderate subgroups based on their HAMA scores, with a threshold of 24 indicating severe anxiety.^33^ Second, the patients were categorized by depressive symptom severity, defined by a cut-off of 24 on the HAMD-17, distinguishing severe from moderate MDD.^34^ Using a consistent analytical approach, we compared group differences in trough duration, SMN time delay, and all FC metrics across HCs and both MDD subgroups.

All statistics, including one-way ANOVA, *t*-tests, and multiple comparison corrections, were conducted using R v4.1.2. The entire analytical process is presented in Supplementary Fig. 1.

## Results

### Clinical profile of patients with A-MDD

During the participant selection process, 15 patients were excluded due to HAMD scores below the threshold of 17. In addition, 17 patients with MDD and 13 HCs were excluded from the analysis due to excessive head-motion artefacts during fMRI scanning (Supplementary Fig. 1). Table 1 presents a summary of the final sample included in the analysis. According to our classification strategy, 44 patients were identified as having A-MDD, while the other 75 patients were identified as having NA-MDD. No difference in age, gender, or education level was found between the two MDD subgroups. However, disparities were noted in gender distribution and education level between HCs and the NA-MDD group. In the clinical scale, we found that total HAMD scores and HAMA scores were greater in the A-MDD group than in the NA-MDD group (*t* = 5.392, *P* < 0.001), (*t* = 4.133, *P* < 0.001). Item-level HAMD scores showed significant group differences in retardation (item 8, *t* = 2.802, *pFDR* = 0.006), psychic anxiety (item 10, *t* = 2.839, *pFDR* = 0.005), appetite decreases (item 12, *t* = 4.919, *pFDR* < 0.001), and sexual interest decreases (item 14, *t* = 3.077, *pFDR* = 0.003) (Supplementary Fig. 2).

**Table 1:** Demographic data for participants

	HC	A-MDD	NA-MDD	Statistics
	(*N* = 94)	(*N* = 44)	(*N* = 75)	
**Gender**				
female	33 (35.1%)	34 (77.3%)	57 (76.0%)	χ^2^ = 36.78 *P* < 0.001 a
male	61 (64.9%)	10 (22.7%)	24 (33.0%)	
**Age**				*F*(2,210) = 0.67, *P* = 0.512
Mean (SD)	34.4 (11.3)	34.9 (12.3)	32.6 (13.0)	
**Education**				
Illiterate	0 (0%)	1 (2.3%)	0 (0%)	*F*(2,209) = 3.89, *P* = 0.022 b
Primary education	4 (4.3%)	3 (6.8%)	5 (6.7%)	
Junior high school	10 (10.6%)	5 (11.4%)	10 (13.3%)	
Senior high school	16 (17.0%)	7 (24.8%)	15 (20.0%)	
Undergraduate	38 (40.4%)	25 (56.8%)	43 (57.3%)	
Graduate	26 (27.7%)	3 (6.8%)	1 (1.3%)	
Missing	0 (0%)	0 (0%)	1 (1.3%)	
**HAMD**				
Mean (SD)	\	30.8 (6.13)	25.2 (5.09)	*t* = 5.392, *P* < 0.001
Median [Min,Max]	\	32 [17, 41]	25 [17, 36]	
**HAMA**				
Mean (SD)	\	24.8 (7.17)	19.7 (6.22)	*t* = 4.133, *P* < 0.001
Median[Min,Max]	\	25 [7, 38]	19 [5, 35]	

*Note.* In the parenthesis, the percentage represents the proportion out of the total for a specific column. Abbreviations: HAMD, Hamilton depression rating scale; HAMA, Hamilton Anxiety rating scale; SD, standard deviation; A-MDD: depression patient with significant agitation symptom; NA-MDD: depression patient without significant agitation.

^a^Pair-wise comparison shows a significant difference between HC and two subgroups.

^b^Pair-wise comparison shows a significant difference between HC and NA-MDD.

### Dynamic brain changes in patients with A-MDD

**Figure 2:**
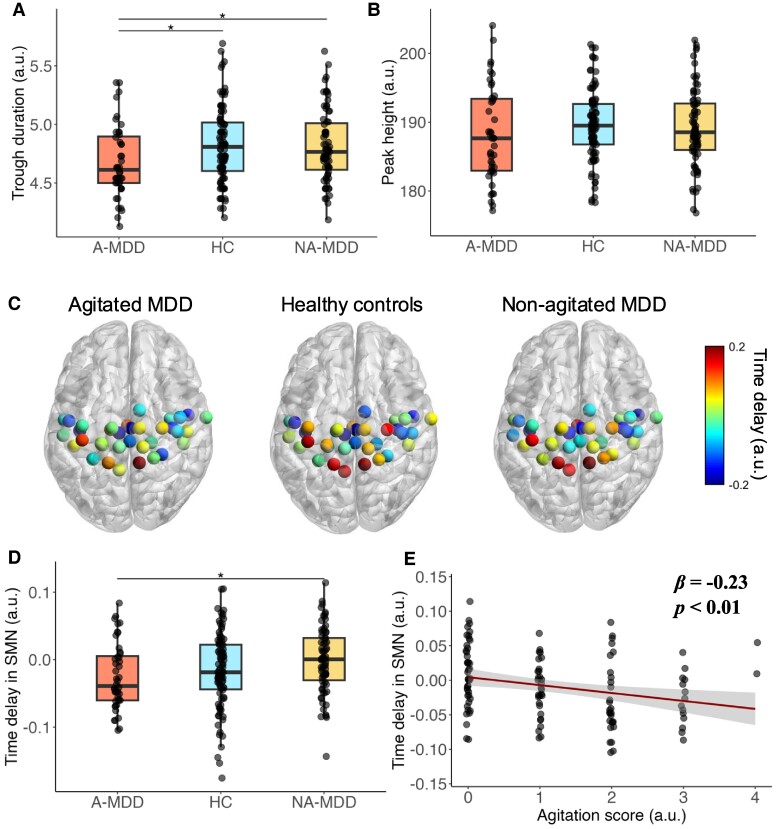
**Dynamic alterations in A-MDD. A, B.** Temporal properties among patients with A-MDD, NA-MDD, and HCs assessed by trough-to-trough duration and peak height. **C**. Illustration of the time delay (TD) estimation in the SMN under Power’s 264 parcellation. Positive and negative values represent early and late propagation, respectively. **D**. Differential TD in the SMN among the three groups. **E**. The association between TD in the SMN and the severity of agitation. This relationship is quantified by a linear regression model, where *β* represents the coefficient of TD in the SMN as a predictor of agitation severity. The model controls for age, gender, and anxiety levels. Agitation scores were derived from the Hamilton Depression Rating Scale. Multiple *t*-tests were performed to estimate group differences in panels **A**, **B**, and **D** for a divergent covariable control strategy. In panels **A**, **B**, and **D**, multiple *t*-tests were conducted to determine group differences, employing a strategy that accounts for divergent covariates. The asterisks in these panels denote statistical significance at the *P* < 0.05 level after FDR correction. Abbreviations: MDD, major depressive disorder.

Overall, we found a different co-fluctuation structure in patients with A-MDD. Specifically, we found that the mean trough duration was narrowed in the A-MDD group compared with the NA-MDD group (*t* = −2.184, *P* = 0.031, *p*FDR = 0.046; Fig. 1A) and HCs (*t* = −2.225, *P* = 0.026, *p*FDR = 0.046; Fig. 2A). No significant difference in trough duration was seen between the NA-MDD and HC groups (*t* = 0.311, *P* = 0.756, *p*FDR = 0.756). By contrast, we did not find significant differences in peak height, which is not a dynamic metric, between any group (A-MDD versus HC: *t* = −0.833, *P* = 0.406, *pFDR* = 0.528; A-MDD versus NA-MDD: *t* = 0.934, *P* = 0.095, *pFDR* = 0.285; NA-MDD versus HC: *t* = 0.001, *P* = 1.00, *pFDR* = 1.00; Fig. 2B).

TD estimation in the SMN was significantly earlier in the A-MDD group than in the NA-MDD group (*t* = −2.616, *P* = 0.011, *p*FDR = 0.031; Fig. 2C and D). However, no significant difference was found in A-MDD versus HC (*t* = −0.263, *P* = 0.793, *p*FDR = 0.793) or NA-MDD versus HC (*t* = −1.551, *P* = 0.123, *p*FDR = 0.185).

However, a significant correlation was found between TD in the SMN and agitation severity. Specifically, the earlier the TD in the SMN, the more severe the agitation (*β* = −0.228, *t* = −2.03, *P* = 0.011; Fig. 2E). However, we did not find a significant correlation between trough duration and agitation symptoms (*β* = −0.012, *t* = −1.36, *P* = 0.177).

### Inter-network connectivity abnormality in patients with A-MDD

**Figure 3:**
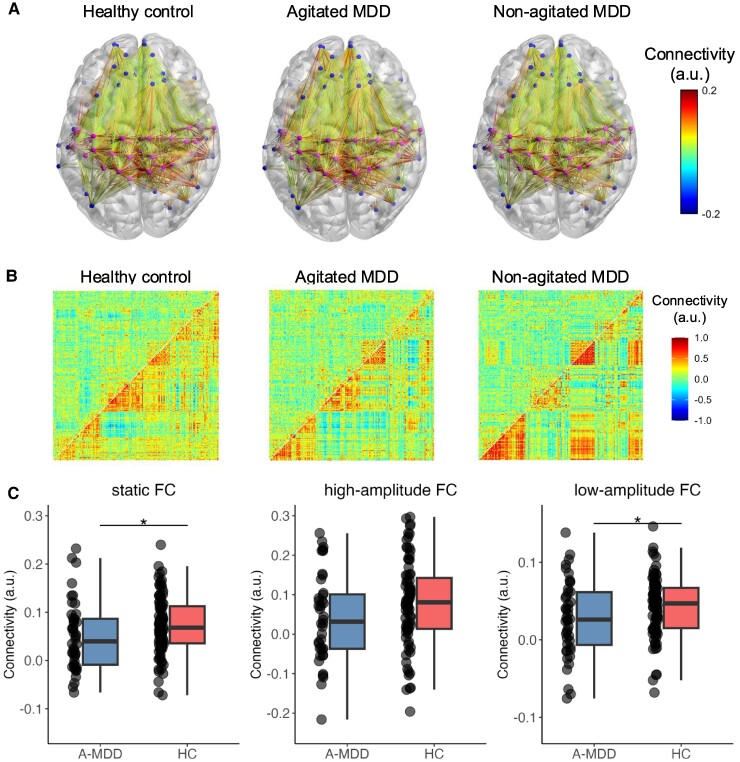
**Inter-network connectivity in different groups. A.** Inter-network connections between the SMN and the DMN. The dots on the pre- and postcentral gyrus represent areas belonging to the SMN, while the dots on the frontal and parital lobe represent areas belonging to the DMN. The colour bar indicates the strength of the connectivity. **B**. Time-varying FC, with the upper triangle showing low-amplitude FC and the lower triangle showing high-amplitude FC. **C**. Static (left), high-amplitude (middle), and low-amplitude FC (right) SMN–DMN connection contrasts between patients with A-MDD and HC. The asterisk indicates the significance of the *t*-value in the *t*-test (* *P* < 0.05, uncorrected). Connectivity was measured by Pearson’s correlation. Abbreviations: MDD, major depressive disorder.

Figure 3 shows the inter-network connectivity alteration in A-MDD. The static SMN–DMN connection was lower in the A-MDD group than in HCs (*t* = −2.102, *P* = 0.037, *pFDR* = 0.111). We identified a decrease in low-amplitude SMN–DMN connections in the A-MDD group compared with the HCs (*t* = −2.139, *P* = 0.034, *pFDR* = 0.102) but not in high-amplitude connections (*t* = 1.671, *P* = 0.097, *pFDR* = 0.291). By contrast, no difference in static SMN–DMN connections was found in NA-MDD versus HC or A-MDD versus NA-MDD (NA-MDD versus HC: *t* = −1.454; A-MDD versus NA-MDD: *t* = −0.067, all *P* > 0.05). Similarly, no difference was found in low- or high-amplitude SMN–DMN connections between the NA-MDD and HC groups (high-amplitude FC: *t* = −0.985; low-amplitude FC: *t* = −0.162, all *P* > 0.05), or between the A-MDD and NA-MDD groups (high-amplitude FC: *t* = −0.421; low-amplitude FC: *t* = −0.298, all *P* > 0.05).

### Re-grouping control analyses

In anxiety-based grouping strategy, no significant differences were found in trough duration in the three groups (severe anxiety MDD versus HC: *t* = −1.04, *P* = 0.451; moderate anxiety MDD versus HC: *t* = −1.18, *P* = 0.451; severe versus moderate anxiety MDD: *t* = 0.03, *P* = 0.947; Supplementary Fig. 3A), or TD in the SMN (severe anxiety MDD versus HC: *t* = 1.26, *P* = 0.601; moderate anxiety MDD versus HC: *t* = 0.48, *P* = 0.635; severe versus moderate anxiety MDD: *t* = 0.84, *P* = 0.601; Supplementary Fig. 3B). In addition, analysis according to depression severity presented non-significant differences in trough durations (severe MDD versus HC: *t* = 0.29, *P* = 0.771; moderate MDD versus HC: *t* = 1.75, *P* = 0.083; severe versus moderate MDD: *t* = −1.75, *P* = 0.082; Supplementary Fig. 3C) and TD in the SMN (severe MDD versus HC: *t* = 0.29, *P* = 0.771; moderate MDD versus HC: *t* = 1.75, *P* = 0.083; severe versus moderate MDD: *t* = −1.75, *P* = 0.082; Supplementary Fig. 3D).

We observed no significant differences in connectivity between the SMN and the DMN in depression severity subgroup analysis, regardless of whether static FC or time-varying FCs were considered (as detailed in Supplementary Table 2). However, when examined by anxiety severity, a significant reduction in low-amplitude FC was identified in the severe anxiety group compared with HCs (*t* = −2.259, *pFDR* = 0.037) and the moderate anxiety group (*t* = −2.045, *pFDR* = 0.011; Supplementary Fig. 4). In addition, a decrease in connectivity in the severe anxiety group was noted in both static FC (*t* = −2.352, *pFDR* = 0.059; Supplementary Fig. 4) and high-amplitude FC (*t* = −2.405, *pFDR* = 0.051; Supplementary Fig. 4) compared with the HCs.

## Discussion

In the present study, we investigated abnormalities in brain dynamics associated with agitation in patients diagnosed with depression. Thirty-seven per cent of the patients exhibiting moderate agitation symptoms were identified as having A-MDD. Our findings, derived from TD estimations and eTS, revealed significant changes in brain dynamics in patients with A-MDD. Specifically, the trough duration of eTS was shorter in those with A-MDD, and TD in the SMN occurred earlier in patients with A-MDD than in patients with NA-MDD, with a negative correlation with agitation severity. In addition, we observed a reduction in SMN–DMN connections in patients with A-MDD. This decrease in connectivity was evident at low-amplitude time points in the eTS. By contrast, no such effects were observed in MDD subgroups defined by depression or anxiety severity.

In our dataset, 37% of the patients were categorized as having A-MDD, with no significant demographic differences observed in patients with A-MDD or non-agitated MDD. Meanwhile, patients with A-MDD exhibited higher HAMD scores than those with NA-MDD, which was indicative of more severe depressive symptoms. In addition, A-MDD was characterized by higher levels of retardation and anxiety. These findings are consistent with previous research, which estimated the prevalence of A-MDD in patients with depressive disorder to be between 34% and 40%,^24,35^ and associated it with the increased severity of both depressive and anxiety symptoms.^36^ Notably, A-MDD is also marked by significant psychomotor retardation, characterized by slowed movements, which, when combined with agitation, presents a complex clinical condition of mixed psychomotor disturbances.^37,38^ Collectively, this symptomatic profile underscores the severity of A-MDD within the spectrum of clinical depression.

We used eTS to evaluate neural co-fluctuations and found a reduction in the duration between successive low-co-fluctuation phases in A-MDD. The temporal property evaluated by eTS represents co-fluctuations across different brain areas, which can be interpreted as transitions between distinct brain states. In this context, the peak-to-trough period represents switching between states.^21^ Previous studies have shown that the state transition rate altered in depression using dynamic functional connectivity (FC).^39,40^ Our findings indicated a rapid transition of brain states in patients with A-MDD, an accelerated neural activity that corresponds to the behavioural symptom manifestations of this subtype. In the framework of STPP, this observed alteration in brain dynamics may signify a discordance between the patient’s subjective temporal perception and their interaction with the external environment, thereby manifesting as the characteristic psychiatric symptoms.^41,42^

We found that TD in the SMN differed between the A-MDD and NA-MDD groups, and the early time lag in the SMN was related to severe agitation. Meanwhile, the connectivity between the SMN and the DMN was reduced in the A-MDD group. Notably, a significant reduction in low-amplitude connections was specifically observed, implying that the aberrant SMN–DMN connection is subject to temporal fluctuations. These findings, concentrating on the SMN, underscore its critical role in psychomotor disturbances in patients with depression. SMN dysfunction in patients with agitated depression (A-MDD) may be attributed to the aberrant transmission of dopamine and serotonin.^43^ The substantia nigra, which projects to the SMN and salience network, has been implicated in the pathophysiology of mood disorders.^43,44^ Disruptions in the dopaminergic system are known to precipitate manic episodes.^45^ Conio *et al*.^46^ showed that the serotonin-related raphe nucleus, which connects with both the SMN and the DMN, indicates a potential interplay between the dopamine and serotonin systems that may underlie the observed temporal shifts in the SMN.

Several limitations should be noted in our study. First, agitation was evaluated based on a 5-point HAMD item score, which only provides a rough description of the symptoms. The CORE questionnaire developed by Parker and McCraw^47^ can assess psychomotor disturbances from a more comprehensive perspective. Given the excellent psychometric attributes of the CORE questionnaire,^47^ future studies could use this measurement to obtain more information on symptoms. Second, temporal fMRI resolution is limited compared with the speed of neuronal propagation. Consequently, the effect size may have been constrained by this limitation, as well as by the small sample size in the current study. Future studies could improve the detection of neuronal transmission by electroencephalography or magnetoencephalography. Finally, alterations in the connectivity between the SMN and the DMN were found in the subgroup divided by anxiety severity. This finding suggests that the functional coupling between these networks is not exclusively associated with agitation. The connectivity between cortical and subcortical regions may offer a potential means to differentiate the unique patterns associated with anxiety and agitation. This hypothesis is supported by previous research. A meta-analysis demonstrated that amygdala-related connectivity was linked to anxiety symptoms,^48^ while another study associated thalamic connectivity with psychomotor disturbances.^49^ Thus, future studies could investigate the connectivity-based markers of agitation within a broader connectome context.

## Conclusion

In this study, we delved into the neural substrates of A-MDD, with a particular focus on the dynamics of brain activity. Our findings revealed significant alterations in brain dynamics among individuals with A-MDD, notably a shortened duration between co-fluctuation troughs, indicative of an accelerated state transition rate. We also observed that fluctuations in the SMN propagated earlier in A-MDD, and this advanced timing was linked to severe agitation. In addition, the connectivity between the SMN and the DMN was diminished in A-MDD, particularly during low-amplitude time points, signifying a directional shift in connectivity patterns. Collectively, our results bolster the STPP hypothesis and contribute to a deeper comprehension of the psychomotor subtype within the spectrum of major depressive disorders.

## Supplementary Material

fcae425_Supplementary_Data

## Data Availability

The extracted fMRI signals, the time delay projection maps for each patient and the codes used in the present study are freely available on an online repository (https://github.com/QunjunLIANG/agitatedMDD). The raw images used in the present study are available from the corresponding author upon resealable request.

## References

[1] Institute for Health Metrics and Evaluation (IHME) . GBD Results. Seattle, WA: IHME, University of Washington; 2024. https://vizhub.healthdata.org/gbd-results/ (link is external).

[2] Williams LM . Precision psychiatry: A neural circuit taxonomy for depression and anxiety. Lancet Psychiatry. 2016;3(5):472–480. 27150382 10.1016/S2215-0366(15)00579-9 PMC4922884

[3] Leventhal AM, Gelernter J, Oslin D, Anton RF, Farrer LA, Kranzler HR. Agitated depression in substance dependence. Drug Alcohol Depend. 2011;116(1-3):163–169. 21277711 10.1016/j.drugalcdep.2010.12.012 PMC3105217

[4] Hasler G, Drevets WC, Manji HK, Charney DS. Discovering endophenotypes for major depression. Neuropsychopharmacology. 2004;29(10):1765–1781. 15213704 10.1038/sj.npp.1300506

[5] Parker G, Paterson A. Melancholia: Definition and management. Curr Opin Psychiatry. 2014;27(1):1–6. 24270479 10.1097/YCO.0000000000000024

[6] Iwanami T, Maeshima H, Baba H, et al Psychomotor agitation in major depressive disorder is a predictive factor of mood-switching. J Affect Disord. 2015;170:185–189. 25248024 10.1016/j.jad.2014.09.001

[7] Benazzi F . Symptoms of depression as possible markers of bipolar II disorder. Prog Neuropsychopharmacol Biol Psychiatry. 2006;30(3):471–477. 16427176 10.1016/j.pnpbp.2005.11.016

[8] Teng X, Ma M, Yang J, Blohm S, Cai Q, Tian X. Constrained structure of ancient Chinese poetry facilitates speech content grouping. Curr Biol. 2020;30(7):1299–1305.e7. 32142700 10.1016/j.cub.2020.01.059 PMC7141976

[9] Kösem A, Bosker HR, Takashima A, Meyer A, Jensen O, Hagoort P. Neural entrainment determines the words we hear. Curr Biol. 2018;28(18):2867–2875.e3. 30197083 10.1016/j.cub.2018.07.023

[10] Northoff G . The brain's spontaneous activity and its psychopathological symptoms—“spatiotemporal binding and integration”. Prog Neuropsychopharmacol Biol Psychiatry. 2018;80(Pt B):81–90. 28363766 10.1016/j.pnpbp.2017.03.019

[11] Lu X, Zhang J-f, Gu F, et al Altered task modulation of global signal topography in the default-mode network of unmedicated major depressive disorder. J Affect Disord. 2022;297:53–61. 34610369 10.1016/j.jad.2021.09.093

[12] Northoff G . Is schizophrenia a spatiotemporal disorder of the brain's resting state? World Psychiatry. 2015;14(1):34–35. 25655148 10.1002/wps.20177 PMC4329887

[13] Northoff G . Are object relations temporal? From the brain’s intrinsic neural timescales over temporo-spatial alignment to object relations. Neuropsychoanalysis. 2022;24(1):47–49.

[14] Northoff G, Magioncalda P, Martino M, Lee H-C, Tseng Y-C, Lane T. Too fast or too slow? Time and neuronal variability in bipolar disorder—A combined theoretical and empirical investigation. Schizophr Bull. 2018;44(1):54–64. 28525601 10.1093/schbul/sbx050 PMC5768053

[15] Ngo GH, Khosla M, Jamison K, Kuceyeski A, Sabuncu MR. Predicting individual task contrasts from resting-state functional connectivity using a surface-based convolutional network. Neuroimage. 2022;248:118849. 34965456 10.1016/j.neuroimage.2021.118849 PMC10155599

[16] He T, Kong R, Holmes AJ, et al Deep neural networks and kernel regression achieve comparable accuracies for functional connectivity prediction of behavior and demographics. Neuroimage. 2020;206:116276. 31610298 10.1016/j.neuroimage.2019.116276 PMC6984975

[17] Zhang L, Zhao J, Zhou Q, et al Sensory, somatomotor and internal mentation networks emerge dynamically in the resting brain with internal mentation predominating in older age. Neuroimage. 2021;237:118188. 34020018 10.1016/j.neuroimage.2021.118188

[18] Cui X, Ding C, Wei J, et al Analysis of dynamic network reconfiguration in adults with attention-deficit/hyperactivity disorder based multilayer network. Cereb Cortex. 2021;31(11):4945–4957. 34023872 10.1093/cercor/bhab133

[19] Ragothaman A, Mancini M, Nutt JG, et al Motor networks, but also non-motor networks predict motor signs in Parkinson’s disease. Neuroimage Clin. 2023;40:103541. 37972450 10.1016/j.nicl.2023.103541 PMC10685308

[20] Martino M, Magioncalda P, Huang Z, et al Contrasting variability patterns in the default mode and sensorimotor networks balance in bipolar depression and mania. Proc Natl Acad Sci U S A. 2016;113(17):4824–4829. 27071087 10.1073/pnas.1517558113 PMC4855585

[21] Zamani Esfahlani F, Byrge L, Tanner J, Sporns O, Kennedy DP, Betzel RF. Edge-centric analysis of time-varying functional brain networks with applications in autism spectrum disorder. Neuroimage. 2022;263:119591. 36031181 10.1016/j.neuroimage.2022.119591 PMC12403185

[22] Mitra A, Snyder AZ, Hacker CD, Raichle ME. Lag structure in resting-state fMRI. J Neurophysiol. 2014;111(11):2374–2391. 24598530 10.1152/jn.00804.2013 PMC4097876

[23] Akiskal HS, Benazzi F, Perugi G, Rihmer Z. Agitated “unipolar” depression re-conceptualized as a depressive mixed state: Implications for the antidepressant-suicide controversy. J Affect Disord. 2005;85(3):245–258. 15780694 10.1016/j.jad.2004.12.004

[24] Benazzi F . Agitated depression: A valid depression subtype? Prog Neuropsychopharmacol Biol Psychiatry. 2004;28(8):1279–1285. 15588754 10.1016/j.pnpbp.2004.06.018

[25] Mitra A, Raichle ME, Geoly AD, Kratter IH, Williams NR. Targeted neurostimulation reverses a spatiotemporal biomarker of treatment-resistant depression. Proc Natl Acad Sci U S A. 2023;120(21):e2218958120. 37186863 10.1073/pnas.2218958120 PMC10214160

[26] Esteban O, Markiewicz CJ, Blair RW, et al fMRIPrep: A robust preprocessing pipeline for functional MRI. Nat Methods. 2019;16(1):111–116. 30532080 10.1038/s41592-018-0235-4 PMC6319393

[27] Power JD, Cohen AL, Nelson SM, et al Functional network organization of the human brain. Neuron. 2011;72(4):665–678. 22099467 10.1016/j.neuron.2011.09.006 PMC3222858

[28] Raut RV, Snyder AZ, Mitra A, et al Global waves synchronize the brain's functional systems with fluctuating arousal. Sci Adv. 2021;7(30):eabf2709. 34290088 10.1126/sciadv.abf2709 PMC8294763

[29] Watanabe T, Rees G. Brain network dynamics in high-functioning individuals with autism. Nat Commun. 2017;8:16048. 28677689 10.1038/ncomms16048 PMC5504272

[30] Raut RV, Mitra A, Snyder AZ, Raichle ME. On time delay estimation and sampling error in resting-state fMRI. Neuroimage. 2019;194:211–227. 30902641 10.1016/j.neuroimage.2019.03.020 PMC6559238

[31] Zamani Esfahlani F, Jo Y, Faskowitz J, et al High-amplitude cofluctuations in cortical activity drive functional connectivity. Proc Natl Acad Sci U S A. 2020;117(45):28393–28401. 33093200 10.1073/pnas.2005531117 PMC7668041

[32] Fleck MP, Poirier-Littre MF, Guelfi JD, Bourdel MC, Loo H. Factorial structure of the 17-item Hamilton depression rating scale. Acta Psychiatr Scand. 1995;92(3):168–172. 7484192 10.1111/j.1600-0447.1995.tb09562.x

[33] Thompson E . Hamilton rating scale for anxiety (HAM-A). Occup Med (Lond). 2015;65(7):601. 26370845 10.1093/occmed/kqv054

[34] Zimmerman M, Martinez JH, Young D, Chelminski I, Dalrymple K. Severity classification on the Hamilton depression rating scale. J Affect Disord. 2013;150(2):384–388. 23759278 10.1016/j.jad.2013.04.028

[35] Benazzi F . Family history validation of a definition of mixed depression. Compr Psychiatry. 2005;46(3):159–166. 16021584 10.1016/j.comppsych.2004.07.034

[36] Parker G, McClure G, Paterson A. Melancholia and catatonia: Disorders or specifiers? Curr Psychiatry Rep. 2015;17(1):536. 25417594 10.1007/s11920-014-0536-y

[37] Benazzi F . A tetrachoric factor analysis validation of mixed depression. Prog Neuropsychopharmacol Biol Psychiatry. 2008;32(1):186–192. 17804137 10.1016/j.pnpbp.2007.08.005

[38] Benazzi F . Defining mixed depression. Prog Neuropsychopharmacol Biol Psychiatry. 2008;32(4):932–939. 18234411 10.1016/j.pnpbp.2007.12.019

[39] Javaheripour N, Colic L, Opel N, et al Altered brain dynamic in major depressive disorder: State and trait features. Transl Psychiatry. 2023;13(1):261. 37460460 10.1038/s41398-023-02540-0 PMC10352359

[40] Xu M, Zhang X, Li Y, et al Identification of suicidality in patients with major depressive disorder via dynamic functional network connectivity signatures and machine learning. Transl Psychiatry. 2022;12(1):383. 36097160 10.1038/s41398-022-02147-x PMC9467986

[41] Northoff G . Spatiotemporal psychopathology II: How does a psychopathology of the brain's resting state look like? Spatiotemporal approach and the history of psychopathology. J Affect Disord. 2016;190:867–879. 26071797 10.1016/j.jad.2015.05.008

[42] Northoff G . Spatiotemporal psychopathology I: No rest for the brain’s resting state activity in depression? Spatiotemporal psychopathology of depressive symptoms. J Affect Disord. 2016;190:854–866. 26048657 10.1016/j.jad.2015.05.007

[43] Northoff G, Hirjak D, Wolf RC, Magioncalda P, Martino M. All roads lead to the motor cortex: Psychomotor mechanisms and their biochemical modulation in psychiatric disorders. Mol Psychiatry. 2021;26(1):92–102. 32555423 10.1038/s41380-020-0814-5

[44] Zhang Y, Larcher KM, Misic B, Dagher A. Anatomical and functional organization of the human substantia nigra and its connections. eLife. 2017;6:e26653. 28826495 10.7554/eLife.26653 PMC5606848

[45] Mukherjee S, Coque L, Cao JL, et al Knockdown of clock in the ventral tegmental area through RNA interference results in a mixed state of mania and depression-like behavior. Biol Psychiatry. 2010;68(6):503–511. 20591414 10.1016/j.biopsych.2010.04.031 PMC2929276

[46] Conio B, Martino M, Magioncalda P, et al Opposite effects of dopamine and serotonin on resting-state networks: Review and implications for psychiatric disorders. Mol Psychiatry. 2020;25(1):82–93. 30953003 10.1038/s41380-019-0406-4

[47] Parker G, McCraw S. The properties and utility of the CORE measure of melancholia. J Affect Disord. 2017;207:128–135. 27721186 10.1016/j.jad.2016.09.029

[48] Zugman A, Jett L, Antonacci C, Winkler AM, Pine DS. A systematic review and meta-analysis of resting-state fMRI in anxiety disorders: Need for data sharing to move the field forward. J Anxiety Disord. 2023;99:102773. 37741177 10.1016/j.janxdis.2023.102773 PMC10753861

[49] Wuthrich F, Lefebvre S, Mittal VA, et al The neural signature of psychomotor disturbance in depression. Mol Psychiatry. 2024;29(2):317–326. 38036604 10.1038/s41380-023-02327-1 PMC11116107

