# Supplementary material for: Temporal dysregulation of the somatomotor network in agitated depression
Source: Brain Commun. 2024 Nov 26;6(6):fcae425. doi: 10.1093/braincomms/fcae425 (PMC11630518; doi:10.1093/braincomms/fcae425)
Supplement: fcae425_Supplementary_Data [file fcae425_supplementary_data.docx]

**Temporal Dysregulation of the Somatomotor Network in Agitated Depression**

**Supplementary Figures**

**Supplementary Figure 1 Schematic Overview of the Analytical Process**

| 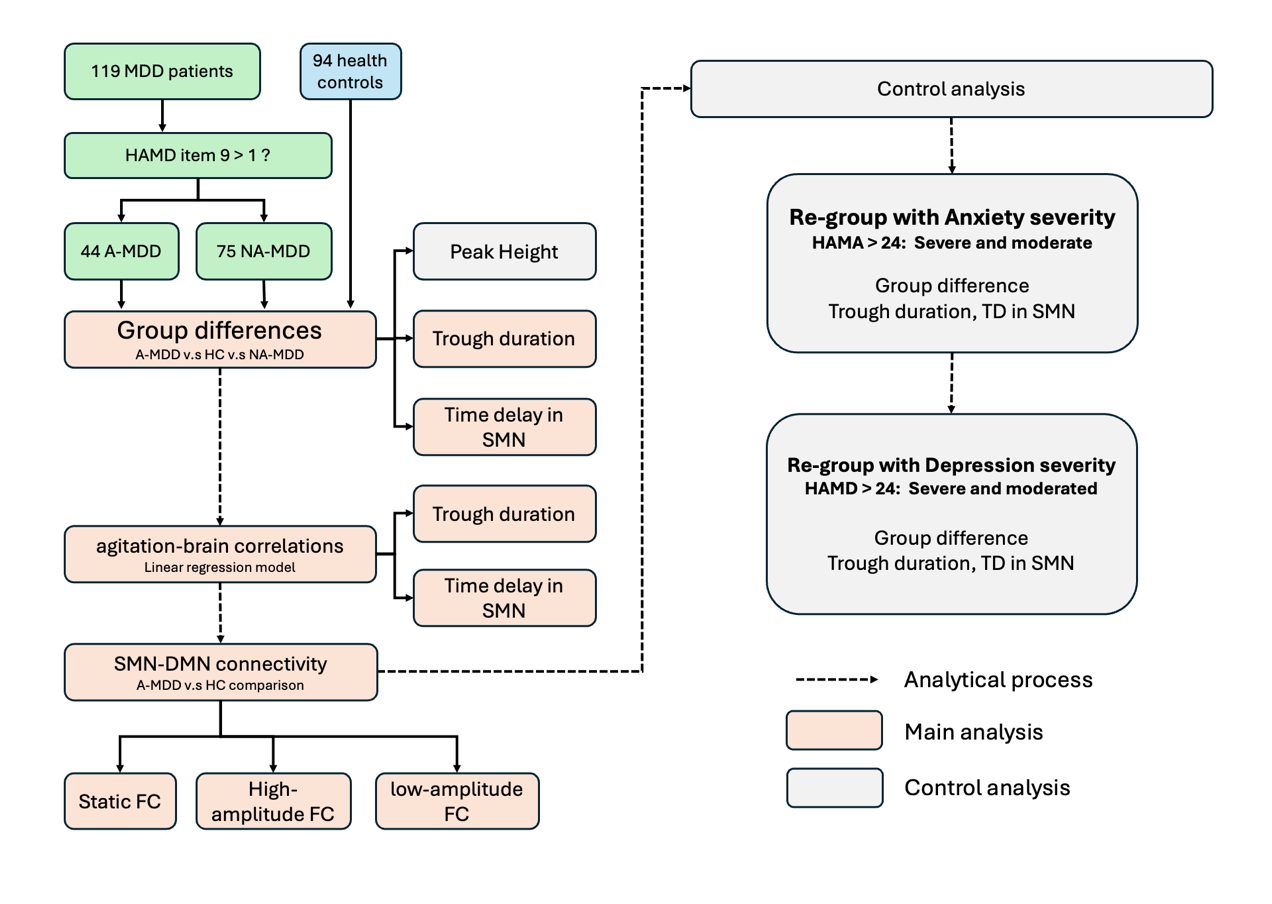 |
| --- |
| **Supplementary Figure 1 Schematic Overview of the Analytical Process.** This schematic delineates the participant grouping strategies and analytical methodologies employed in the study. The primary analysis is compartmentalized into three distinct segments: (1) Examination of group differences in brain dynamic metrics, (2) Correlation analysis between dynamic metrics and symptomatology, and (3) Assessment of brain connectivity differences. For the control analysis, the identical testing protocols were implemented, yet with varied grouping strategies, to ascertain whether the observed effects are specific to the agitation subgroup. |

**Supplementary Figure 2 Symptom specific between agitation and non-agitation patients**

| 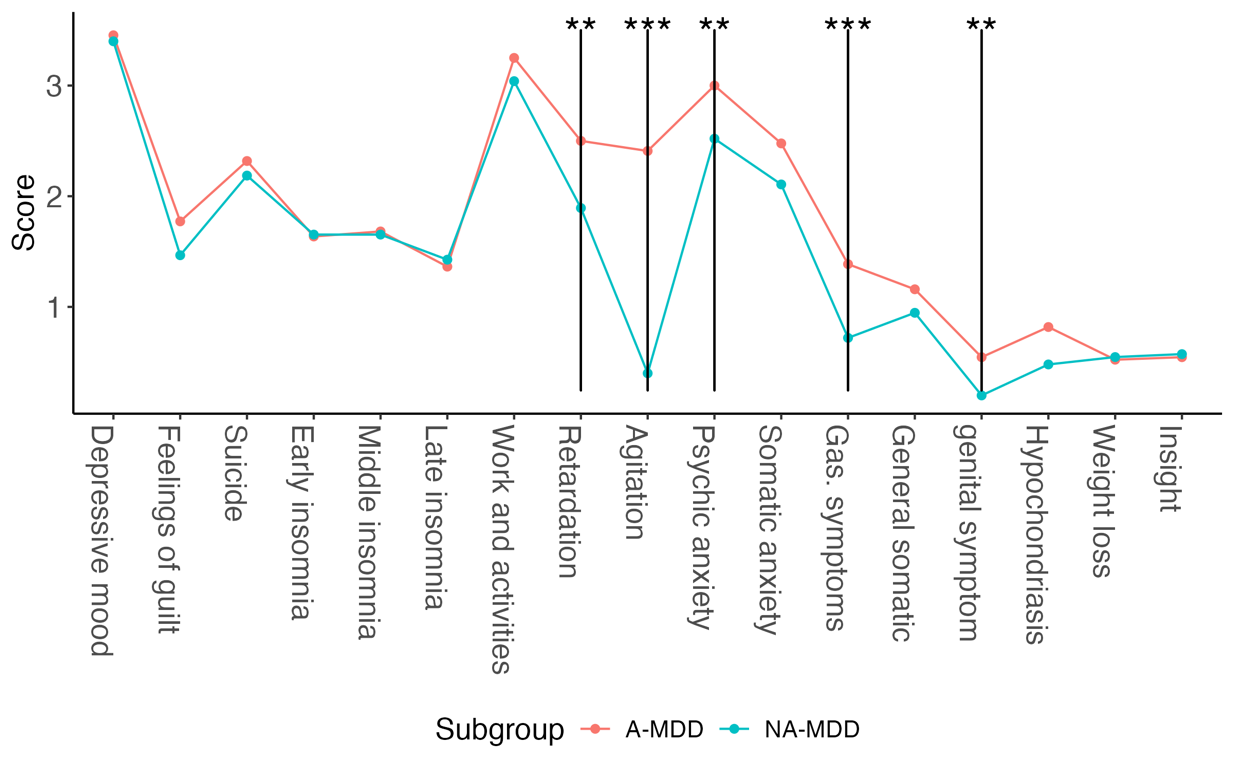 |
| --- |
| **Supplementary Figure 2 Symptom specific between agitation and non-agitation patients.** This figure displays the symptom various between two subgroups of patients. The symptoms were evaluated using the 17-item Hamilton Rating Scale for Depression (HAMD-17). The x-axis represents the symptom corresponding to each item in HAMD-17, while the y-axis indicates the mean score for each subgroup. * *p* < .05, ** *p* < .001, *** *p* < .001. |

**Supplementary Figure 3 Brain dynamic difference in re-grouping strategies**

| 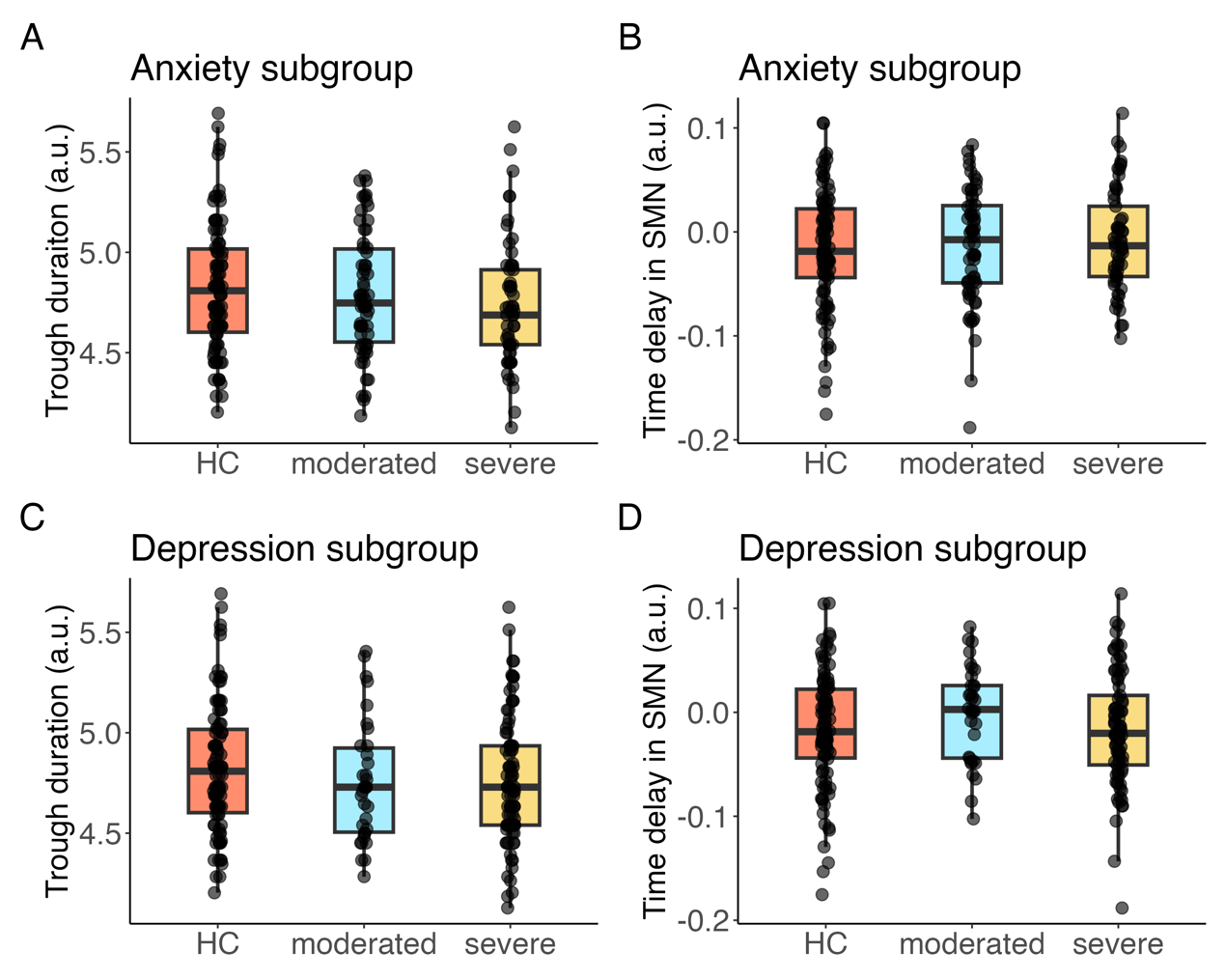 |
| --- |
| **Supplementary Figure 3 Brain dynamic difference in re-grouping strategies.** (A) Depicts the group difference in trough duration between healthy controls (HC) and the collective cohort of patients diagnosed with Major Depressive Disorder (MDD). (B) Illustrates the group difference in the time delay of the somatomotor network (SMN) between HC and MDD. (C) Demonstrates the group difference in trough duration when comparing HC to the stratified MDD subgroups, categorized as moderate and severe. (D) Presents the group difference in the time delay of SMN among HC and the differentiated MDD subgroups, delineated by severity. |

**Supplementary Figure 4 Functional connectivity differences within the anxiety severity grouping strategy**

| 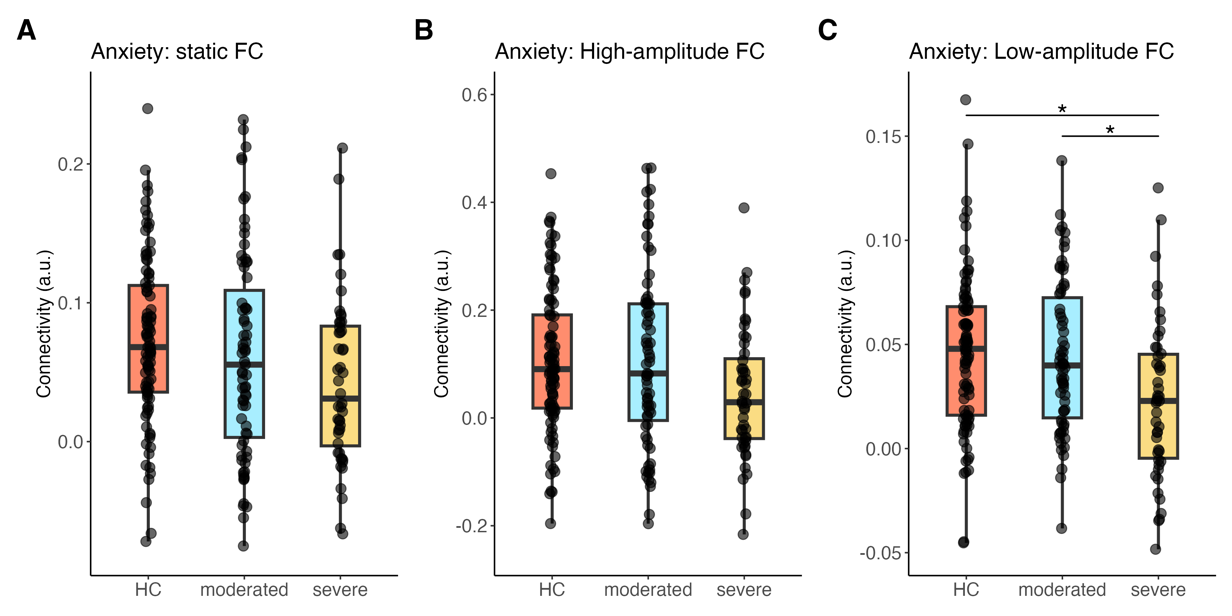 |
| --- |
| **Supplementary Figure 4 Functional connectivity differences within the anxiety severity grouping strategy.** Panels illustrate the group comparison among healthy controls (HC), severe and moderated anxiety subgroups for (A) static functional connectivity (FC), (B) high- and (C) low-amplitude time-varying FCs. The comparison was performed by using t-tests. * *p* < 0.05, after FDR correction. |

**Supplementary Tables**

**Supplementary Table 1 Recruited participants’ information**

|  | HC (n = 107) | MDD (n = 151) |
| --- | --- | --- |
| **Age** |  |  |
| Mean (SD) | 35.1 (11.8) | 33.5 (12.7) |
| **Gender** |  |  |
| Female | 33 (30.8%) | 110 (72.8%) |
| Male | 74 (69.2%) | 41 (27.2%) |
| **Education** |  |  |
| Illiterate | 0 (0%) | 2 (1.3%0 |
| Primary education | 4 (3.7%) | 10 (6.6%) |
| Junior high school | 16 (15%) | 16 (10.6%) |
| Senior high school | 20 (18.7%) | 30 (19.9%) |
| Undergraduate | 41 (38.3%) | 85 (56.3%) |
| Graduate | 26 (24.3%) | 4 (2.6%) |
| Missing | 0 (0%) | 4 (2.6%) |
| **HAMD** |  |  |
| Mean (SD) | / | 25.6 (7.05) |
| Median [Min, Max] | / | 26 [8, 41] |
| **HAMA** |  |  |
| Mean (SD) | / | 20.5 (7.27) |
| Median [Min, Max] | / | 20 [5, 38] |
| *Note.* In the parenthesis, the percentage represents the proportion out of the total for a specific column. Abbreviations: HAMD, Hamilton depression rating scale; HAMA, Hamilton Anxiety rating scale; SD, standard deviation. | | |

**Supplementary Table 1 Group comparisons within depression severity grouping strategy for SMN-DMN connectivity**

| **Contrast estimate** | **t-value** | ***p*FDR** | **Cohen’s d** |
| --- | --- | --- | --- |
| *Static FC* | | | |
| Severe vs. moderated MDD | -0.467 | 0.641 | 0.096 |
| Severe MDD vs. HC | -1.949 | 0.158 | 0.327 |
| Moderated MDD vs. HC | -1.082 | 0.421 | 0.231 |
| *High-amplitude FC* | | | |
| Severe vs. moderated MDD | -0.603 | 0.547 | 0.124 |
| Severe MDD vs. HC | -1.562 | 0.361 | 0.262 |
| Moderated MDD vs. HC | -0.647 | 0.547 | 0.138 |
| *Low-amplitude FC* | | | |
| Severe vs. moderated MDD | 0.711 | 0.478 | 0.146 |
| Severe MDD vs. HC | -1.579 | 0.174 | 0.265 |
| Moderated MDD vs. HC | -1.922 | 0.168 | 0.411 |
| *Abbreviations*: FC, functional connectivity; MDD, major depressive disorder; HC, healthy controls; FDR, false-discovery rate. | | | |
